# Persistent Traumatic Stress Exposure: Rethinking PTSD for Frontline Workers

**DOI:** 10.3390/healthcare14020255

**Published:** 2026-01-20

**Authors:** Nicola Cogan

**Affiliations:** Department of Psychological Sciences and Health, Graham Hills Building, University of Strathclyde, Glasgow G11XQ, UK; nicola.cogan@strath.ac.uk

**Keywords:** Persistent Traumatic Stress Exposure (PTSE), trauma-informed practice, organisational readiness, organisational preparedness, psychological safety, frontline workers, workforce mental health, cumulative

## Abstract

**Highlights:**

**What are the main findings?**

- Persistent Traumatic Stress Exposure (PTSE) is introduced as a framework for understanding cumulative and ongoing trauma in frontline work, synthesising evidence linking repeated exposure, moral challenges, and organisational pressures to psychological distress.
- Psychological distress among frontline workers is shaped not only by discrete traumatic events, but by sustained occupational exposure embedded within organisational contexts.
- Trauma exposure is identified as a shared, cross-sector workforce challenge rather than an issue confined to specific roles or services.

**What are the implications of the main findings?**

- Preventative approaches should prioritise trauma-informed systems, organisational readiness, and preparedness to protect workforce mental health.
- Workforce mental health strategies need to move beyond individualised responses to incorporate structural and organisational interventions.
- Coordinated, whole-system approaches to trauma-informed readiness and preparedness are required across frontline sectors to mitigate cumulative psychological harm.

**Abstract:**

Frontline workers across health, emergency, and social care sectors are repeatedly exposed to distressing events and chronic stressors as part of their occupational roles. Unlike single-event trauma, these cumulative exposures accrue over time, generating persistent psychological and physiological strain. Traditional diagnostic frameworks, particularly post-traumatic stress disorder (PTSD), were not designed to capture the layered and ongoing nature of this occupational trauma. This commentary introduces the concept of Persistent Traumatic Stress Exposure (PTSE), a framework that reframes trauma among frontline workers as an exposure arising from organisational and systemic conditions rather than solely an individual disorder. It aims to reorient understanding, responsibility, and intervention from a purely clinical lens toward systems, cultures, and organisational duties of care. PTSE is presented as an integrative paradigm informed by contemporary theory and evidence on trauma, moral injury, organisational stress, and trauma-informed systems. The framework synthesises findings from health, emergency, and social care settings, illustrating how repeated exposure, ethical conflict, and institutional pressures contribute to cumulative psychological harm. PTSE highlights that psychological injury may build across shifts, careers, and lifetimes, requiring preventive, real-time, and sustained responses. The framework emphasises that effective support is dependent on both organisational readiness, the structural conditions that enable trauma-informed work, and organisational preparedness, the practical capability to enact safe, predictable, and stigma-free responses to trauma exposure. PTSE challenges prevailing stigma by framing trauma as a predictable occupational hazard rather than a personal weakness. It aligns with modern occupational health perspectives by advocating for systems that strengthen psychological safety, leadership capability and access to support. By adopting PTSE, organisations can shift from reactive treatment models toward proactive cultural and structural protection, honouring the lived realities of frontline workers and promoting long-term wellbeing and resilience.

## 1. Introduction

When does trauma ever become post?” This question, posed by a paramedic during our research on workplace trauma [1], captures the paradox at the heart of frontline reality—one I have encountered repeatedly in both clinical practice and research. Across frontline and public-facing occupations, trauma is rarely a discrete incident that can be left behind. For many frontline and public-facing workers, exposure is cumulative, recurrent, and embedded within the organisational context of their role. It becomes woven into daily work, accumulating shift by shift and across careers [2]. Every call carries the potential for loss or tragedy, and it is the repetition, year after year, that wears down resilience and erodes any “after-event” boundary [3]. Studies describe cumulative burden and the impossibility of closing the chapter on distress [4,5,6]. Large-scale data show that repeated duty-related incidents and organisational stressors predict PTSD-like trajectories more reliably than single events [7]. Yet, the diagnostic category of PTSD continues to assume a clear “before” and “after.” For frontline workers, there is often no “after.” Existing diagnostic frameworks are not designed to fully capture this persistent exposure. Most were designed to describe trauma as a discrete event rather than a continuous occupational reality. This mismatch matters: when our language does not reflect lived experience, support systems, and organisational practices risk missing the mark. Persistent Traumatic Stress Exposure (PTSE) is therefore proposed as a reframing, positioning trauma not as an individual disorder but as an occupational hazard, emphasising prevention, systemic responsibility, and the potential for resilience and growth among those who serve our communities.

This commentary is informed by my clinical and academic work within the NHS and the third sector, alongside research conducted with frontline workers and sustained engagement with peer support workers and individuals with lived experience. Across these contexts, recurring patterns of cumulative exposure, organisational strain, and moral challenge were evident. Repeatedly encountered in my clinical and research work, these patterns informed the development of PTSE as a complementary framework that extends existing diagnostic understandings by foregrounding organisational and systemic dimensions of trauma exposure.

## 2. The Limitations of PTSD for Frontline Workers

The PTSD diagnosis, introduced in 1980 following the Vietnam War [8], was ground-breaking in recognising that trauma can have profound and enduring psychological consequences. Although not limited to single incidents, early formulations largely conceptualised trauma in relation to identifiable stressors and post-event symptom patterns, with less attention to cumulative, occupational, or organisational exposure. For its time, this gave long-overdue legitimacy to experiences that had previously been minimised or dismissed. Over time, diagnostic frameworks evolved: DSM-5 expanded symptom clusters, and ICD-11 introduced Complex PTSD to reflect prolonged interpersonal adversity [9]. Yet they were not originally designed to capture the occupational ecology of frontline and public-facing professionals, where trauma emerges through cumulative exposure embedded within professional identity. Although DSM-5 expanded Criterion A to include repeated or extreme indirect exposure to aversive details of trauma, this expansion still operates within an event-based framework. The requirement for identifiable traumatic exposure means that occupational trauma is often conceptualised in relation to discrete events or exposures, rather than as a continuous condition of work embedded within organisational contexts. This distinction is crucial. For many frontline workers, the psychological burden arises not only from repeated encounters with Criterion A-level events but also from the enduring occupational environment that sustains hypervigilance, ethical strain, organisational pressure, emotionally demanding interactions, and chronic uncertainty. These forms of exposure do not fit neatly within Criterion A, yet they are well-established predictors of poor mental health and burnout. The persistence of exposure, rather than the frequency of discrete events, is what characterises frontline trauma. This conceptual gap between the definitional scope of Criterion A and the lived experience of frontline work is arguably where PTSE adds both conceptual and practical value.

Contemporary research reinforces this picture. PTSD is now understood as one of several trajectories shaped by exposure burden, resilience processes, and organisational context [10,11]. Frontline workers consistently report high rates of PTSD symptoms, depression, burnout, and suicidality [12,13,14]. In the United States, suicide rates among firefighters and police officers exceed those of military veterans [15]. In the United Kingdom, nearly half of intensive care staff met criteria for a probable mental disorder during the 2020–2021 COVID-19 surge, with rates remaining close to 45% thereafter [16]. Among Scottish health and social care workers, 49.3% scored above the clinical cut-off for acute stress [17]. Longitudinal studies show that repeated exposure and organisational stressors, rather than isolated incidents, shape symptom pathways and recovery [18]. While PTSD remains an essential diagnostic framework, it is not always well aligned with the lived realities of frontline and public-facing professionals. Event-based models are less well suited to capturing forms of trauma that are continuous and embedded within occupational life. PTSE offers a complementary lens that illuminates the cumulative hazards and burdens borne by those who keep our communities safe. Taken together, this literature suggests the need for a framework that sits alongside diagnostic models, one that enables cumulative occupational exposure and organisational context to be conceptualised without collapsing them into individual pathology.

## 3. Introducing Persistent Traumatic Stress Exposure (PTSE)

In response to this gap, PTSE is proposed as a complementary framework for understanding cumulative occupational exposure alongside existing diagnostic models. PTSE reframes trauma as an occupational exposure rather than an individual disorder, shifting attention from personal vulnerability to the structural and organisational conditions of work. The notion of persistence recognises that trauma is not confined to a single moment but accumulates across shifts, careers, and lifetimes. The emphasis on exposure invites us to think about trauma in the same way other occupational hazards are understood. Just as asbestos exposure is addressed through regulation, monitoring, and protective equipment, trauma exposure can be recognised as a systemic risk that requires collective strategies of protection and prevention. At the same time, it is important to acknowledge that, unlike physical hazards such as asbestos, traumatic stress is inherently more nuanced and contextual. In frontline settings, the impact of repeated exposure is shaped not only by frequency or intensity but also by organisational culture, leadership responses, moral context, the availability of psychological safety, and individuals’ own personal histories. As such, PTSE does not imply a mechanistic model of harm; rather, it emphasises how cumulative exposure interacts with relational, systemic, and personal factors to shape outcomes. This framing retains the utility of the occupational hazard analogy while remaining grounded in the lived realities of frontline work.

In frontline clinical settings, PTSE is shaped by sustained exposure to patient and care complexity, which provides an important empirical anchor for the framework. Across frontline services, patient complexity reflects a cumulative burden of medical instability, psychosocial vulnerability, continuous complex decision-making, and responsibility for unpredictable outcomes, accruing across shifts rather than through single events. Evidence shows that higher levels of patient and nursing complexity independently predict adverse outcomes, such as intra-hospital transfer and intensive care admission, even when controlling for clinical severity [19]. From a PTSE perspective, what becomes particularly salient is how sustained exposure to complex care environments entails ongoing vigilance, ethical and moral strain, and responsibility for deterioration and risk. This pattern supports understanding PTSE as a system-embedded occupational exposure, shaped by organisational context and role demands, rather than as a solely psychological phenomenon.

Emerging research lends support to this perspective. For example, ref. [20] found that firefighters move dynamically between distress and growth, resisting simple categorisations of illness or resilience. Outcomes are shaped by resilience processes and repeated exposure, challenging the linear assumptions often implied by traditional PTSD frameworks [6]. Research on moral injury also highlights how betrayal by systems and ethically compromising environments create lasting psychological consequences that extend beyond individual events [21]. Together, these findings point towards PTSE as a valuable addition to the trauma lexicon, one that better reflects the occupational ecology of frontline workers and acknowledges both risk and the possibility of post-traumatic growth (see Table 1).

Framing trauma as persistent exposure rather than an episodic disorder has important implications for how organisations conceptualise risk, allocate resources, and discharge their duty of care. An exposure-based PTSE framework encourages approaches that attend to cumulative burden, role-related risk, and system pressures, rather than relying solely on individual symptom thresholds or post hoc diagnosis. This perspective may support earlier recognition of risk, clearer organisational accountability, and more equitable access to support for workers whose distress arises from sustained occupational demands rather than discrete traumatic events. In this way, PTSE offers a practical lens for informing staffing practices, leadership approaches, and preventive investment, aligning psychological safety with established principles of occupational health and risk management.

## 4. Implications for Practice, Policy, and Culture

Recognising PTSE calls for a reorientation in how trauma among frontline and public-facing professionals is understood and addressed. Clinically, it challenges reactive, diagnosis-driven models that wait for distress to crystallise before care is offered. Longitudinal studies demonstrate that early symptoms often predict worsening outcomes when left untreated [22]. During the COVID-19 pandemic, almost one-third of healthcare workers met criteria for probable PTSD, with distress persisting long after the acute phase of the crisis had passed [23,24]. Trauma must therefore be approached as a predictable occupational hazard, requiring early, preventive, and embedded interventions rather than episodic, post hoc responses.

Evidence indicates that the most effective programmes are proactive and culturally embedded, normalising help-seeking and peer support within organisational life [25,26,27]. Innovations in digital tools co-designed with frontline workers provide real-time support for stress regulation and cumulative exposure, complementing clinical care and embedding mental health protection into everyday practice in much the same way as physical safety procedures [28,29]. Once trauma is recognised as exposure rather than individual vulnerability, responsibility shifts from the worker to the system [30]. Employers across the emergency, health, and social care sectors therefore hold a duty to mitigate psychological risk in the same way they manage physical hazards [31]. The concept of *PPE for the mind*, articulated by a frontline professional in our research, captures this responsibility by framing wellbeing checks, peer networks, psychologically literate supervision, and compassionate leadership as core components of occupational risk management rather than discretionary supports [32].

Culturally, PTSE reframes trauma as exposure rather than weakness, helping to dismantle stigma and legitimise help-seeking in professions where identity is often closely tied to toughness and endurance. Despite growing awareness, stigma and fear of being perceived as unfit for duty remain significant barriers to support [33]. Many of the inequities experienced by frontline workers can be understood through the lens of structural stigma, referring to the policies, diagnostic frameworks, and organisational practices that shape how trauma is recognised, legitimised, and responded to [34]. Within frontline settings, dominant PTSD classifications can inadvertently reinforce structural stigma by privileging discrete, diagnosable events over cumulative occupational exposure, thereby limiting recognition, protection, compensation, and access to support for those whose distress arises from persistent conditions of work. By adopting an exposure-based lens, PTSE aligns with public health approaches to suicide prevention that move beyond individual blame and towards collective responsibility [35] while also acknowledging how perceived institutional betrayal and moral injury can amplify distress [36,37]. In doing so, PTSE clarifies inequities embedded within prevailing trauma classifications and strengthens the case for equitable protection, monitoring, and resourcing across roles and sectors, aligning psychological harm with other regulated workplace risks.

A PTSE framework also has implications for the future development of AI-enabled and digital mental health interventions. When trauma is understood as cumulative and occupational, scalable and adaptive approaches are required to address inequities embedded within event-based diagnostic and support systems. Evidence suggests that AI-supported interventions, when co-created with frontline workers and key stakeholders, can support earlier identification of cumulative risk and widen access to timely support, thereby helping to address structural stigma. Critically, participatory co-creation is essential to ensure that such technologies reflect lived experience, occupational realities, and ethical priorities, and do not reproduce existing systemic biases within algorithmic systems [38,39].

Finally, PTSE invites reconsideration of post-traumatic growth within occupational contexts characterised by ongoing exposure. For many frontline workers, trauma does not end, and growth cannot be meaningfully framed as something that occurs “after” adversity. Instead, growth may be more accurately understood as sustained functioning, adaptation, and meaning-making under persistent exposure. In practice, this may be reflected in the development of greater emotional literacy, more attuned risk assessment, strengthened peer solidarity, and a deepened sense of professional purpose despite continued exposure to distress. Some frontline workers describe growth not as transformation following trauma, but as the ability to remain engaged, compassionate, and ethically grounded over time. Others report shifts in boundaries, values, and leadership practices that support both self-preservation and team functioning. Framed in this way, growth becomes a dynamic and ongoing process embedded within systems of psychological safety and organisational support, rather than an individual outcome that presumes resolution of trauma. This reconceptualisation aligns more closely with frontline realities and situates post-traumatic growth within the PTSE framework as a collective and occupational phenomenon rather than an endpoint [40]. A next step in developing PTSE is the development and validation of a psychometric measure, work that I am currently undertaking with a multidisciplinary research team that includes frontline professionals with lived experience of occupational trauma and expertise in psychological safety. This measure is being examined in relation to existing measures of acute stress, trauma exposure, and moral injury, to support empirical testing and refinement of the framework.

## 5. The Human and Systemic Cost of Ignoring PTSE

When PTSE goes unrecognised, the consequences are profound. Firefighters repeatedly exposed to traumatic incidents often withdraw from family life. Call handlers haunted by voices of those they could not save carry those memories home. Police officers may turn to alcohol or other substances to dull intrusive memories. Healthcare and social care staff working through unrelenting crises experience compassion fatigue, depersonalisation, and burnout [41]. These are not isolated personal struggles but signs of a systemic failure to protect workers from predictable occupational hazards.

The impact extends beyond the workplace. Rising levels of quiet quitting and sickness absence reflect how cumulative exposure erodes motivation, health, and empathy [42]. Partners and children often absorb the emotional residue of trauma, navigating irritability, withdrawal, or emotional numbing in their loved ones. The home becomes a secondary site of exposure, where stress reverberates through family dynamics, leading to marital strain and intergenerational effects [43]. The erosion of wellbeing ripples outward, undermining families, organisations, and the resilience of communities [44]. Addressing PTSE requires more than awareness; it demands a reconfiguration of care and responsibility. Trauma must be treated as an entrenched occupational hazard that calls for longitudinal and preventive approaches. Proactive, embedded interventions consistently outperform reactive models. Organisations should integrate psychological safety into daily operations, treating PPE for the mind [45] as essential as physical protection, while societies must acknowledge trauma as inseparable from occupational duty.

Researchers, clinicians, and policymakers must collaborate to capture the lived realities of exposure and embed psychological support within workplace culture. Trauma prevention should be viewed as a shared responsibility comparable to physical risk management [46]. Valuing frontline workers means moving beyond rhetoric toward sustained protection and compassion. Recognising PTSE provides a foundation for a systemic framework of care, one that reduces harm, strengthens resilience, and fosters growth, ensuring those who serve our communities are not only safeguarded but supported to thrive [47].

## 6. Trauma-Informed Systems: Readiness and Preparedness

Trauma-informed systems are characterised not only by an awareness of trauma but by the capacity to respond safely, consistently, and with organisational coherence [48]. Within this literature, a useful distinction has emerged between readiness and preparedness. Readiness refers to the structural conditions that enable trauma-informed work, including policies, leadership capability, psychological safety, shared language, and cultural principles. Preparedness concerns the practical ability to enact those conditions in everyday contexts, such as recognising trauma exposure, responding appropriately, and ensuring that routes to support are accessible, confidential, and non-stigmatising. This distinction is highly relevant to PTSE, in which staff are not affected by a single critical incident but by recurrent and cumulative exposure. In such environments, emotional strain becomes normalised, and organisations may underestimate the degree of impact precisely because trauma exposure is routine. PTSE therefore calls for an organisational response that goes beyond episodic critical-incident management and toward ongoing relational and systemic support. It is the interaction between readiness and preparedness that enables staff to remain psychologically safe in the face of repeated stress.

Recent work shows that many organisations express philosophical commitment to trauma-informed principles, yet structures, pathways, and leadership practices are often underdeveloped or inconsistently applied [49]. This creates conditions in which staff may continue to function, yet do so under sustained pressure, embedded within systems that offer limited recognition or support. Organisational readiness without preparedness risks remaining symbolic, while preparedness is difficult to sustain without the infrastructure, policies, and leadership behaviours that constitute readiness.

Importantly, PTSE also foregrounds the need for a whole-society approach to trauma-informed readiness and preparedness. Frontline exposure does not occur in isolation within single organisations or sectors; health, social care, emergency services, defence, justice, transport, and other critical infrastructures are increasingly interdependent, particularly during periods of national or global crisis. Pandemics, major incidents, climate-related emergencies, and conflict generate simultaneous psychological demands across multiple systems, often requiring coordinated responses between civilian and defence services. In this context, preparedness cannot be understood solely at the level of individual organisations. Trauma-informed readiness must also be conceptualised as a shared societal capacity, supported by aligned leadership principles, interoperable support pathways, and consistent expectations regarding psychological safety and duty of care across sectors. Without such alignment, frontline workers may move between systems with markedly different levels of protection, support, and cultural norms, compounding cumulative exposure and inequity.

In the context of PTSE, trauma-informed systems therefore require both relational and structural conditions at organisational and societal levels: clear communication, active and psychologically literate leadership, predictable responses, safe mechanisms for disclosure, and opportunities for ongoing learning and reflection. Embedding these principles at scale involves moving beyond isolated interventions towards systematic cultural and organisational change, where persistent exposure to trauma is recognised as an occupational reality rather than an individual problem, and where support is integrated within everyday practice and across interconnected frontline systems.

## 7. Conclusions

This commentary positions PTSE as a complementary lens for understanding cumulative occupational trauma in frontline work. Informed by clinical practice, research with frontline workers, and sustained engagement with peer support and lived experience, PTSE responds to patterns of distress that emerge through ongoing exposure rather than discrete traumatic events. While PTSD remains invaluable for clinical recognition and treatment, it was not originally designed to capture the organisational, cultural, and contextual conditions through which trauma is repeatedly encountered in frontline and public-facing roles.

Conceptualising trauma as a persistent occupational exposure rather than an individual vulnerability shifts attention from personal pathology to collective responsibility. This reframing reflects realities observed across frontline services, where distress often arises through cumulative exposure, moral challenge, and sustained responsibility rather than singular incidents. PTSE therefore offers a way of validating lived experience, reducing self-blame, and supporting more equitable recognition of frontline trauma, while remaining aligned with established diagnostic approaches and their essential clinical role.

PTSE also foregrounds the importance of readiness and preparedness in shaping outcomes under persistent exposure. Where exposure is predictable, and organisations demonstrate clear leadership, psychological safety, and accessible support pathways, frontline workers may sustain functioning, adaptability, and ethical engagement despite ongoing demands. In this context, growth is understood not as an outcome that follows trauma, but as sustained functioning supported by trauma-informed systems rather than individual endurance. It points toward a model of trauma understanding that is clinical, organisational, and public health-oriented in scope, offering a complementary perspective through which cumulative occupational exposure in frontline work may be more clearly recognised and addressed.

## Figures and Tables

**Table 1:** Conceptual distinctions between post-traumatic stress disorder (PTSD) and Persistent Traumatic Stress Exposure (PTSE).

Dimension	Post-Traumatic Stress Disorder (PTSD)	Persistent Traumatic Stress Exposure (PTSE)
Nature of trauma	Discrete, identifiable event(s) often occurring in the past	Continuous, cumulative exposure across shifts, careers, and systems
Temporal framing	“Post”—assumes a before and after the event	“Persistent”—ongoing, with no clear endpoint or recovery boundary
Locus of problem	Individual psychopathology	Occupational and systemic exposure
Primary focus of intervention	Diagnosis and treatment of symptoms	Prevention, mitigation, and systemic protection
Responsibility for management	Individual and clinical services	Shared organizational, cultural, and policy responsibility
Stigma and identity implications	May reinforce vulnerability narratives	Reframes distress as an occupational hazard, reducing stigma
Clinical orientation	Reactive, treatment-focused (after harm)	Proactive, preventive, and embedded within daily work
Analogous model	Medical model (disease or disorder)	Public health and occupational safety model
Potential outcomes	Recovery or chronic PTSD	Resilience, adaptation, and sustained growth within a protective system

## Data Availability

Not applicable. No new data were collected from human participants.
